# 16S rRNA metagenomic data of microbial diversity of *Pheidole decarinata* Santschi (Hymenoptera: Formicidae) workers

**DOI:** 10.1016/j.dib.2020.106037

**Published:** 2020-07-17

**Authors:** Mohammed Ahmed Ashigar, Abdul Hafiz Ab Majid

**Affiliations:** a Department of Zoology, Federal University of Lafia, Nasarawa State, Nigeria; b Household & Structural Urban Entomology Laboratory, Vector Control Research Unit, School of Biological Sciences, Universiti Sains Malaysia, Minden, Penang 11800, Malaysia

**Keywords:** Metagenome, Microbial DNA, *P. decarinata*, Proteobacteria

## Abstract

Metagenomic datasets of the microbial DNA of workers of a *Pheidole decarinata* Santschi (Hymenoptera: Formicidae) around houses with three replicates were presented. Next-generation sequencing of the microbial DNA was performed on an Illumina Miseq platform. QIIME (version 1.9.1) was used to analyze the raw fastq files. Metagenome of the three (3) samples consist of 333,708 sequences representing 137,359,149 bps with an average length of 413.67 bps. The sequence data is available at the NCBI SRA with the bioproject number PRJNA632430. Community analysis revealed Proteobacteria was the predominant (84.77%) microbial community present in the microbial DNA of workers of the *P. decarinata*.

**Specifications Table**

**Table:** 

**Subject**	Biology
**Specific subject area**	Microbiology Genomics
**Type of data**	Table Figure 16S rDNA Illumina sequence
**How data were acquired**	Community metagenome analysis was carriedout after 16S v3-v4 amplicon metagenomics sequencing was performed.
**Data format**	Raw FASTQ files Analyzed
**Parameters for data collection**	Workers of *Pheidole spp* collected in urban settlement
**Description of data collection**	The microbial DNA of the ant samples were extracted from a pooled of crushed minors and majors of *Pheidole decarinata* collected within urban communities of Lafia, Nigeria using HiYield™ Genomic DNA isolation kit (Real Biotech Corporation, Taiwan). 16S (v3-v4) amplicon metagenomic sequencing was performed on Illumina MiSeq platform.
**Data source location**	Institution: Universiti Sains Malaysia City/Town/Region: Lafia Country: Nigeria Latitude and longitude (and GPS coordinates) for collected samples/data: 08°30′08.95″ N 08°31′21.95″ E
**Data accessibility**	Repository name: NCBI SRA Data identification number: PRJNA632430 Direct URL to data: https://www.ncbi.nlm.nih.gov/sra/PRJNA632430

**Value of the Data**

• The data provides microbial diversity of *Pheidole decarinata* collected in urban areas.
• Little or no data exist on the community metagenomic of Pheidole ants commonly observed around residentials and non-residential areas in urban communities.
• The data offers possibility in the discovery of novel bacteria that have not previously been reported from ants.
• The metagenomic data also benefit future comparative and phylogenetic studies of microbial diversity of *P. decarinata*

## 1 Data description

Total sequences of 138,548, 120,456, 74,704 with average read length of 404.18, 409.71, 427.14 base pairs (bps) were produced by the Illumina Miseq sequencer from the samples LGI, LG2 and LG3, respectively as shown in Table 1. LGI, LG2 and LG3 are codes given to the different locations where the samples were collected within Lafia GRA (LG) in Nasarawa, Nigeria.

**Table 1:** Number of sequences, base pairs, and average length of the sequences from *Pheidole decarinata* collected from LG1, LG2 and LG3.

Sample*	Sequences	Bases(bp)	Average Length(bp)
LG1	138,548	56,764,392	409.71
LG2	120,456	48,685,953	404.18
LG3	74,704	31,908,804	427.14

⁎ LGI, LG2 and LG3 are codes given to *Pheidole decarinata* collected from different locations within in Nasarawa, Nigeria. The alphabet LG represents Lafia GRA while the numbers (1,2 and 3) represents the different collection sites (foraging trails/colonies).

**Fig. 1:**
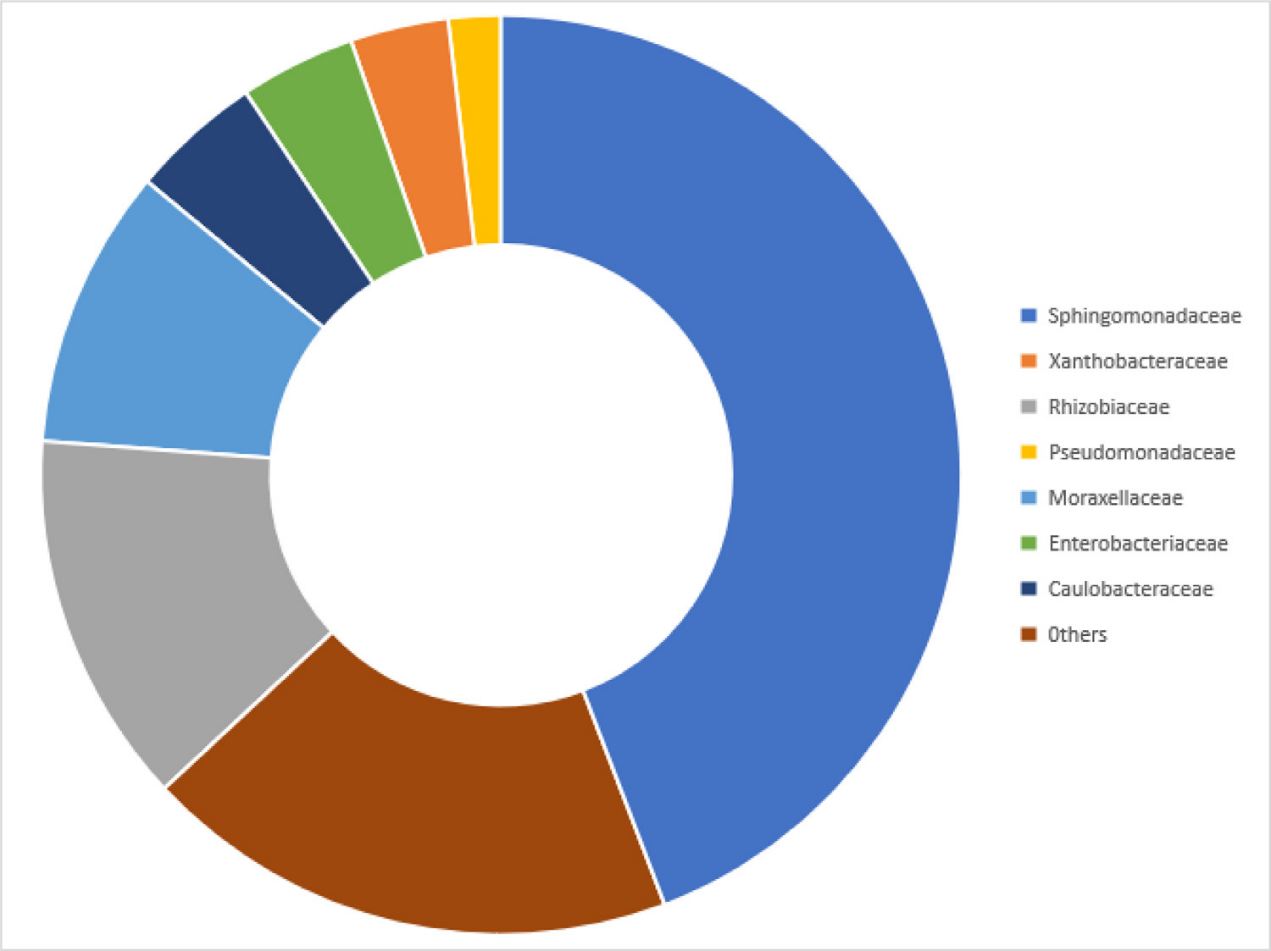
Relative abundance of dominant bacterial families with more than 1% reads in microbiome of the *Pheidole decarinata*.

**Fig. 2:**
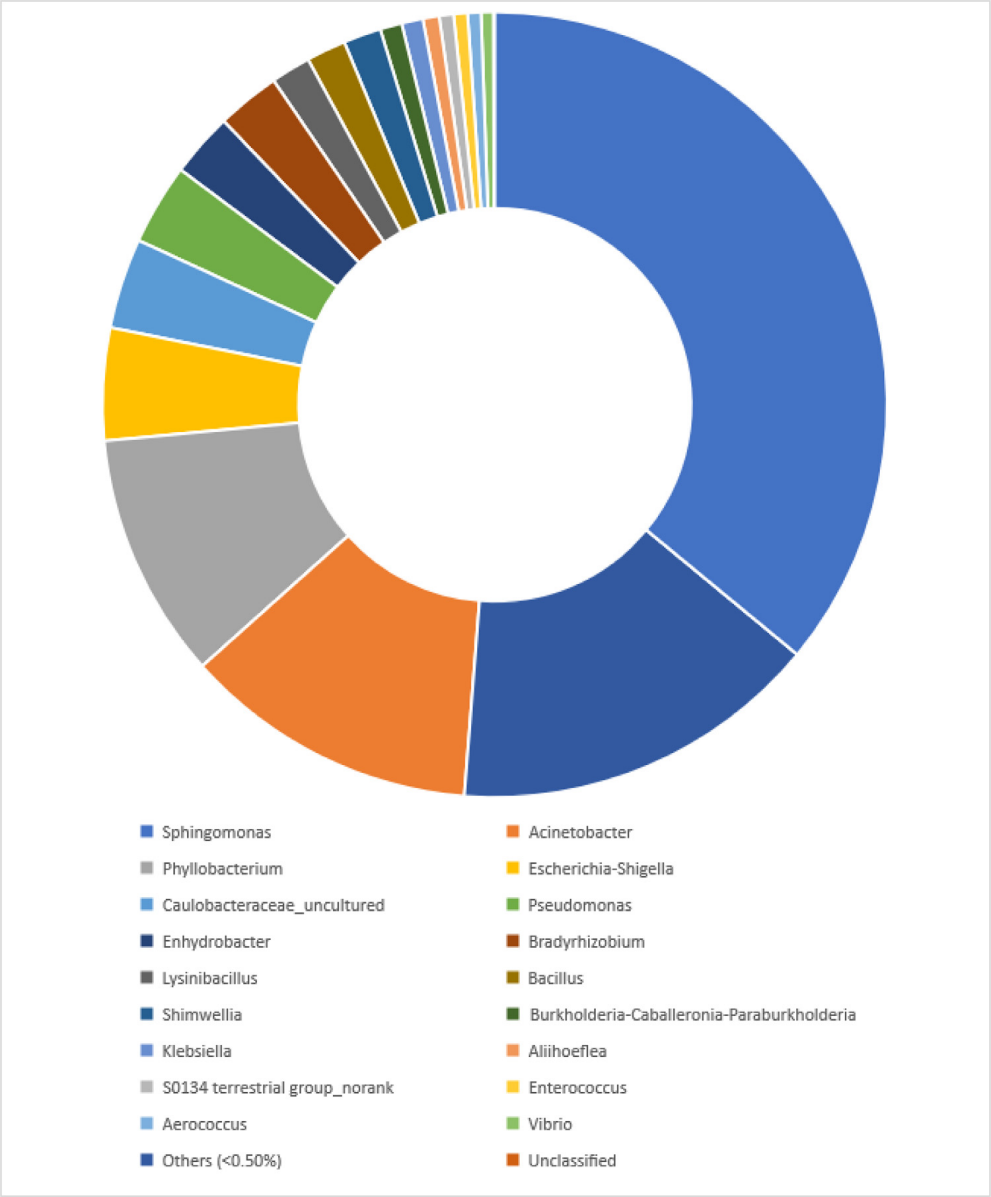
Relative abundance of the dominant bacterial genera with 0.50% and above percentage reads in microbiome of the *Pheidole decarinata*.

Community analysis of the three samples revealed that Proteobacteria (84.77%) was the predominant microbial phylum present in the microbial DNA of workers of a *Pheidole decarinata*. Other phyla present are Firmicutes (6.00%), Actinobacteria (5.11%) and Others (4.13%) with less than 0.50% of the reads. A total of 265 families were recorded in the microbiome and only seven (7) bacterial families had relative abundance of more than 1%. They include Sphingomonadaceae (44.21%), Rhizobiaceae (13.12%), Moraxellaceae (9.85%), Caulobacteraceae (4.61%), Enterobacteriaceae (4.07%), Xanthobacteraceae (3.45%), Pseudomonadaceae (1.84%) and families with less than 1% prevelence were grouped in others (18.85) as shown in Fig. 1. The most prevalent bacterial classes were Alphaproteobacteria, Gammaproteobacteria, Betaproteobacteria and Bacilli (Firmicutes). 36.31% of sequences of the three samples were assigned to the genus Sphingomonas (Alphaproteobacteria), 12.27% to Acinetobacter (Gammaproteobacteria), 10.22% to Phyllobacterium (Alphaproteobacteria), 4.66% to Escherichia-Shigella (Gammaproteobacteria) and percentage reads of other classes and genera with greater than 0.50% were summarized in Fig. 2.

## 2 Experimental design, materials, and methods

### 2.1 Insect sampling

Workers (major and minor) of *P. decarinata* were collected in and around houses in Lafia, Nigeria. The ants were observed foraging households and baited with other insects (American cockroach) then collected into sterile tubes. Each foraging trails was preserved separately and were sorted and identified using standard taxonomic keys. The metagenomic analysis of the ant samples carried out to determine their bacteria composition. The ant samples were collected from different location and *P. decarinata* collected within same trail were pooled prior to DNA extraction [1]. Ten workers (both major and minor) of *P. decarinata* from same trail were used for each replicate. The samples were rinsed several times [2] with sterile distilled water to remove soil and other debris from the samples before further molecular analysis.

### 2.2 DNA extraction

Next-generation sequencing (NGS) was employed to investigate the microbial diversity of *Pheidole decarinata*. The genomic DNA were extracted from the insect samples using HiYield™ Genomic DNA isolation kit (Real Biotech Corporation, Taiwan) according to the manufacturer's protocols with little modifications. The ant specimens were washed with sterile distill water severally to remove soil, plants and other debris attached to the surface of the samples. Ten (10) major and minor workers from same foraging trail (colony) were pooled together and pounded in a 200 μL of 1X PBS with sterile pestle according to [3]. 1X PBS was used to replace QGT Buffer and mixed concurrently with Proteinase K and QGB Buffer before tissue homogenization and incubation. Incubation time was reduced to 2 h [3]. other steps such as DNA binding, washing and elution were done according to the HiYield™ Genomic DNA isolation kit protocols. The little modifications in the DNA isolation procedure was performed to achieve high molecular weight DNA [3]. Three (3) DNA samples (LG1, LG2 and LG3) were extracted from *Pheidole decarinata* were sent to MyTACG Bioscience Enterprise (Kualar Lumpur, Malaysia) for Illumina sequencing.

### 2.3 PCR amplification, amplicons purification and quantification

The V3–V4 marker region of the bacteria were amplified by PCR (95 °C for 2 min, followed by 25 cycles at 95 °C for 30 s, 55 °C for 30 s, and 72 °C for 30 s and a final extension at 72 °C for 5 min) using primers pairs 341F (5′-CCTAYGGGRBGCASCAG-3′) and 806R (5′-GGACTACNNGGGTATCTAAT-3′). PCR reactions were performed in 20 μL mixture containing 4 μL of 5 × FastPfu Buffer, 2 μL of 2.5 mM dNTPs, 0.8 μL of each primer (5 μM), 0.4 μL of FastPfu Polymerase, and 10 ng of template DNA. Amplicons were extracted from 2% agarose gels and purified using the AxyPrep DNA Gel Extraction Kit (Axygen Biosciences, Union City, CA, U.S.) according to the manufacturer's instructions and quantified using QuantiFluor™ -ST (Promega, U.S.).

### 2.4 Library construction and Illumina sequencing

Library construction was done by removing adapters dimer using beads and single-stranded DNA fragments were generated using sodium hydroxide. Sample libraries were pooled in equimolar and paired-end sequenced (2 × 250/300 bp) on an Illumina MiSeq platform according to the standard protocols. Raw fastq files were demultiplexed, quality-filtered using QIIME (version 1.9.1) [4]. Low quality reads with average quality score <20 were trimmed using Trimmomatic software [5] and the trimmed reads with lengths shorter than 50 bp were discarded. Paired-reads were merged to single read using FLASH (Fast Length Adjustment of Short reads) [6] based on overlapped relationship. Reads which could not be assembled were discarded. Operational Units (OTUs) were clustered with 97% similarity cutoff using UPARSE (version 7.1 http://drive5.com/uparse/) [7]. Chimeric sequences were identified and removed using UCHIME [8]. The taxonomy of each 16S rRNA gene sequence was analyzed by RDP Classifier (http://rdp.cme.msu.edu/) [9] against the Silva (SSU123) 16S rRNA database [10] using confidence threshold of 0.7.

## Declaration of Competing Interest

The authors declare that no known competing financial interests or personal relationships which have, or could be perceived to have, influenced the work reported in this article.

## Appendix Supplementary materials

Image, application 1
